# The prevalence and clinical correlation factors of cognitive impairment in patients with major depressive disorder hospitalized during the acute phase

**DOI:** 10.3389/fpsyt.2024.1497658

**Published:** 2024-11-15

**Authors:** Huiyuan Zhao, Jinhong Chen

**Affiliations:** ^1^ Department of Psychiatry, The School of Clinical Medicine, Hunan University of Chinese Medicine, Changsha, Hunan, China; ^2^ Department of Psychiatry, The Second People's Hospital of Hunan Province (Brain Hospital of Hunan Province), Changsha, Hunan, China

**Keywords:** cognitive impairment, major depression disorder, acute phase, clinical correlation factors, hospitalized patients

## Abstract

**Objective:**

This study aimed to investigate the prevalence of cognitive impairment among patients with major depressive disorder (MDD) hospitalized during the acute phase and to analyze the in-depth association between this cognitive impairment and clinical correlation factors.

**Methods:**

In this cross-sectional study, we recruited 126 patients aged between 18 and 65 years who were diagnosed with MDD. All these patients were inpatients from the Department of Psychiatry at the Second People’s Hospital of Hunan Province. We employed a series of assessment tools, including the Pittsburgh Sleep Quality Index (PSQI), the 16-item Dysfunctional Beliefs and Attitudes about Sleep Scale (DBAS-16), the Pre-sleep Arousal Scale (PSAS), the Morningness-Eveningness Questionnaire (MEQ), the Hamilton Anxiety Rating Scale (HAMA), and the 17-item Hamilton Depression Rating Scale (HAMD-17). The patients were divided into a cognitive impairment group and a non-cognitive impairment group based on their scores on the Montreal Cognitive Assessment Scale (MoCA). Through Spearman’s correlation analysis, we explored the correlation between the total MoCA score and the score of each factor. Additionally, we utilized binary logistic regression analysis to investigate the relationship between cognitive impairment and clinically relevant factors in MDD patients hospitalized during the acute phase and plotted ROC curves to evaluate their clinical efficacy.

**Results:**

In this study, we found that the prevalence of cognitive impairment among MDD patients hospitalized during the acute phase was as high as 63.49%. Through statistical analysis, we observed significant differences between the cognitive impairment group and the non-cognitive impairment group in terms of age, place of residence, education level, and HAMD-17 scores. In the Spearman correlation analysis, we noted the following trends: visuospatial and executive abilities were negatively correlated with the HAMD-17 score (*P* < 0.05); naming ability was positively correlated with the PSAS score but negatively correlated with the MEQ score (*P* < 0.05); memory was also negatively correlated with the MEQ score (*P* < 0.05); attention was negatively correlated with the HAMA score; and abstract cognitive ability was negatively correlated with the MEQ score (*P* < 0.05). Through binary logistic regression analysis, we further revealed the relationship between cognitive impairment and factors such as living in a rural area (OR = 2.7, 95% CI = 1.083-6.731, *P* < 0.05), increased age (OR = 1.049, 95% CI = 1.013-1.087, *P* < 0.01), and the HAMD-17 score (OR = 1.10295, 95% CI = 1.031-11.79, *P* < 0.01). Additionally, ROC curve analysis demonstrated a significant correlation between the HAMD-17 score and the prediction of cognitive function in MDD patients hospitalized during the acute phase (*P* < 0.001). Specifically, the AUC for the HAMD-17 score was 0.73, with an optimal cut-off value of 19.5, sensitivity of 70.0%, and specificity of 63.0%. Furthermore, the AUC for age was 0.71, with an optimal cut-off value of 33.5, sensitivity of 59.0%, and specificity of 80.0%.

**Conclusions:**

This study indicates that MDD patients hospitalized during the acute phase have a higher prevalence of cognitive impairment. This phenomenon reflects a significant correlation between clinical factors such as age, sleep-related characteristics, and the severity of depression with cognitive impairment. Therefore, regular assessment of cognitive function in MDD patients and early intervention may be crucial for the treatment and prognosis of the disease.

## 1 Introduction

Major depressive disorder (MDD) is a psychiatric disease characterized by persistent depressive symptoms. Its clinical manifestations also include reduced interest, anhedonia, decreased appetite and libido, disinterest in sexual function, and cognitive impairment (1). In extremely severe cases, patients may develop suicidal thoughts or even engage in suicidal behavior, thereby constituting a serious mental disorder. A systematic review indicated that the global annual cumulative incidence of MDD and depressive symptoms ranged from 3.9% to 4.5% (2) In China, the overall prevalence of MDD among the elderly population aged 60 and above was 24.3%, while in the younger population, this proportion was slightly higher, reaching 28.4% (3, 4). This indicates a trend of earlier onset of MDD, which may impose significant economic pressure on families and society.

In addition to affective symptoms, patients with MDD often experience cognitive decline, manifested as impaired attention, memory, and executive function (5). MDD is not only a mental illness but also a significant risk factor for cognitive decline and dementia, with potentially long-lasting effects on patients. For example, a longitudinal study involving 22,789 participants with a 15-year follow-up showed that baseline depressive symptoms significantly elevated the incidence of dementia and cognitive impairment across the entire sample, especially in the younger population (6). Moreover, another long-term follow-up study found that the frequency of MDD episodes was positively correlated with the risk of dementia. A single depressive episode can increase the risk of dementia by 87%-92%, while two or more episodes almost double the risk (7). These findings have significantly heightened awareness of the profound impact MDD can have on cognitive function.

The incidence of cognitive impairment varies across different stages of MDD. In the prodromal and acute phases, the incidence of cognitive impairment is as high as 76.9% to 94.0%. During the remission period, the proportion decreases from 32.4% to 44.0% (8). Although fluctuations in depressive symptoms are closely related to cognitive function, studies have shown that improvements in cognitive function in patients with depression do not completely align with the relief of affective symptoms. Even with significant or partial relief of affective symptoms, the incidence of cognitive impairment can still reach as high as 44.0%. On the one hand, cognitive impairment during the acute phase may indicate the potential for long-term cognitive issues, making patients more likely to experience recurrent episodes of depression in the future. On the other hand, the suicide risk associated with MDD is significantly higher than that of other mental disorders. According to a review, MDD is a significant risk factor for suicide (9). Previous research has indicated that suicidal thoughts or behaviors in MDD patients may be related to cognitive impairment during the acute phase, and diminished cognitive function may further increase the risk of suicide (10). Among adolescents with MDD, cognitive impairment is particularly pronounced, especially in areas such as inhibitory control, verbal fluency, switching ability, attention, and memory. These cognitive impairments contribute to adolescent impulsivity, poor classroom performance, low self-esteem, poor academic and social adjustment, and even an increased risk of suicidal behavior (11).

Early and effective identification and alleviation of cognitive impairment in patients with MDD are crucial for maximizing clinical recovery rates and significantly reducing the risk of suicide throughout the entire treatment process. Currently, although numerous studies have confirmed that some patients with acute MDD may experience varying degrees of decline in language, attention, working memory, verbal memory, processing speed, and executive function, there is still a lack of effective intervention methods in clinical practice (12). A meta-analysis suggested that most antidepressant drugs primarily target the affective symptoms of MDD, such as sadness, hopelessness, despair, and loss of interest, without demonstrating significant effects in improving cognitive functions such as attention, memory, and decision-making (13). Therefore, effectively identifying risk factors for cognitive decline during the acute phase of MDD treatment may become an important strategy for enhancing cognitive function in depressed patients.

Previous studies have demonstrated a strong correlation between depression, sleep disorders, and cognitive function (14). The primary sleep-related symptoms in patients with MDD include difficulty falling asleep, early morning awakening, frequent nighttime awakenings, and vivid dreaming (15). Polysomnography monitoring has revealed that, compared to healthy individuals, MDD patients exhibit abnormal sleep architecture, characterized by reduced slow-wave sleep (SWS), increased rapid eye movement (REM) sleep, and shortened REM sleep latency (16). The decrease in SWS may lead to the overactivation of central and peripheral immune-inflammatory factors, resulting in excessive phosphorylation of β-amyloid and tau proteins in the brain, which can exacerbate neuronal damage (17). Moreover, insufficient sleep in these patients may lead to decreased levels of brain-derived neurotrophic factor (BDNF) and glial cell line-derived neurotrophic factor (GDNF) (18). BDNF, a protein crucial for neuronal survival, development, differentiation, and repair, plays a key role in regulating neuronal structure, plasticity, synaptic connections, and neurotransmitter transmission. Studies have shown that attention, working memory, and information processing speed in the brain are positively correlated with BDNF levels (19, 20). Another study that collected data on cerebrospinal fluid, cognitive abilities, and sleep quality from 1,168 Alzheimer’s patients revealed that shorter sleep duration (< 7 hours) was significantly associated with higher levels of cerebrospinal fluid t-tau and p-tau (19). In summary, poor sleep quality and low sleep efficiency in MDD patients may be key contributors to cognitive impairment.

The circadian rhythm, commonly referred to as the biological clock, typically describes an endogenous 24-hour periodic rhythm that is prevalent in various biological processes (21). In the human body, this rhythm significantly influences sleep-wake cycles and metabolic processes. Research showed that compared to healthy individuals, patients with MDD experienced significant daytime functional impairments in both positive and negative emotions, with symptoms often being most severe in the early morning (22). In a circadian rhythm analysis involving 60 patients with MDD who were in remission, it was found that 35% of the patients identified as morning types, 58.3% as intermediate types, and 6.7% as evening types. These patients generally felt more fatigued in the morning and struggled to perform complex cognitive tasks (23). This suggests that disruptions to the circadian rhythm can negatively impact cognitive function. However, there is limited evidence to suggest a higher degree of excessive wakefulness and erroneous sleep-related cognitive beliefs in MDD patients. Increased bedtime activities, mental rumination, physical discomfort, and incorrect sleep cognitions may contribute to excessive wakefulness, resulting in circadian rhythm disturbances and sleep disorders, which ultimately exacerbate cognitive impairment (24).

There is currently no consensus on the cognitive function of patients with MDD, which is likely closely related to the characteristics of research objects and the inconsistency of research methods. Previous studies have compared the cognitive performance of MDD patients with different characteristics and identified several factors that may affect cognitive function, including increased levels of tumor necrosis factor, interleukin-8, and macrophage inflammatory protein (MIP)-1β in plasma, as well as an elevated body mass index (BMI). These factors may explain the deficits MDD patients experience in processing speed and working memory (25). Additionally, patients with late-onset MDD exhibited more significant deficiencies in memory, verbal fluency, and language and visuospatial abilities compared to those with early-onset MDD (26). However, there are only a few studies that have conducted in-depth evaluations of cognitive function and its associated factors during the acute phase of MDD, which provides us with further motivation for the exploration of this research area. Based on this, we planned to recruit patients with MDD with the aim of evaluating their cognitive function and examining clinically relevant characteristics such as sleep patterns and anxiety levels. The main objective of this study was to reveal the prevalence of cognitive impairment in MDD patients hospitalized during the acute phase and to analyze in detail the association between these impairments and clinically relevant factors.

## 2 Methods

### 2.1 Study design and participants

This cross-sectional investigation took place between December 2023 and June 2024. Psychiatric hospitalized patients in the Second People’s Hospital in Hunan Province served as the participants.

The inclusion criteria are as follows (1): fulfills the Diagnostic and Statistical Manual of Mental Disorders, Fifth Edition (DSM-V) criteria for MDD; (2) being of Han nationality and aged between 18 and 65; (3) a 17-item Hamilton Depression Rating Scale (HAMD-17) score ≥17 points; (4) patients with first or recurrent depression in the acute phase who are not taking antidepressants or other treatments that affect cognitive function for nearly 2 weeks; (5) cooperating with the completion of questionnaires and psychological evaluation; and (6) signed informed consent.

The exclusion criteria are as follows: (1) having previously suffered from physical illnesses that caused cognitive impairment; (2) previously suffered from mental disorders that led to cognitive impairment; (3) unable to collaborate and communicate regularly; and (4) no signed informed consent form.

A total of 126 participants took part in the study out of the 143 patients who were initially enrolled as 17 patients were excluded for lack of data or other reasons. The questionnaire was administered by three well-trained clinical psychiatrists. The Ethics Review Committee of the Second People’s Hospital of Hunan Province approved this study (No. 2023 (K) 018), and each participant signed a written informed consent form.

### 2.2 Sample size

The sample size was calculated as $N=\frac{z_{\propto /2\;}^{2}P\left(1-p\right)}{\delta^{2}}$ in the study. According to the literature, the frequency of cognitive impairment in patients with MDD during the acute phase ranges from 85% to 94% (27), with *P* = 0.94. Assuming a tolerance error of 5%, we set α = 0.05, which corresponds to $z_{\alpha /2}=1.96$ . Considering potential invalid responses at a rate of 10%, the final required sample size should be adjusted to reach at least 95 cases; thus, this study ultimately included a sample size of 126 cases.

### 2.3 Measurements

Three specially trained clinical psychiatrists conducted a detailed questionnaire survey with all participants to collect sociodemographic data and clinical characteristics. The data collected included information such as age, gender, height, weight, marital status, education level, comorbidities, place of residence, and bed partner. BMI was calculated by dividing body weight (in kg) by the square of height (in m). Based on the BMI values, we classified the patient into the following categories: underweight (BMI < 18.5 kg/m2), normal weight (BMI 18.5-24 kg/m2), overweight (24 ≤ BMI < 28 kg/m2), and obese (≥ 28 kg/m2) (28). After the participants completed the first part of the basic information collection, we proceeded to evaluate other clinically relevant factors.

#### 2.3.1 The Pittsburgh Sleep Quality Index

This scale is suitable for patients with mental or sleep disorders and the general population with the goal of evaluating sleep quality (29). It consists of 19 self-rated items and 5 observer-rated items, primarily divided into seven components: subjective sleep quality, sleep latency, sleep duration, sleep efficiency, sleep disturbances, use of sleep medications, and daytime dysfunction. Each section is scored from 0 to 3 points. The overall PSQI score ranges from 0 to 21 points, which is the sum of the scores for each section. A higher score indicates poorer sleep quality. The overall reliability coefficient for the Chinese version of the PSQI is 0.82 to 0.83 (30).

#### 2.3.2 The 16-item Dysfunctional Beliefs and Attitudes about Sleep Scale

This scale aims to assess sleep-related misconceptions and beliefs, particularly self-perceived sleep misconceptions (31). It consists of 16 questions, rated on a scale from 1 to 5 points, resulting in a total score range of 16 to 80 points. The lower the score, the higher the incidence of erroneous beliefs. The total reliability coefficient for the Chinese version of the DBAS-16 is 0.765 (32).

#### 2.3.3 The Pre-Sleep Arousal Scale

The PSAS comprises two dimensions, i.e., physical and cognitive arousal, specifically designed to assess a patient’s level of wakefulness before bedtime (33). The scale consists of 16 test items, each scored on a 5-point scale ranging from 1 to 5 points. A higher score indicates an improvement in cognitive or physical arousal. The overall score ranges from 16 to 80 points. The overall reliability coefficient of the Chinese version of the PSAS is 0.91 (34).

#### 2.3.4 The Morningness-Eveningness Questionnaire

The MEQ is primarily used to evaluate an individual’s circadian rhythm type, consisting of 19 items (35). The total score ranges from 16 to 86 points. Specifically, a score below 41 indicates a tendency toward an evening type, a score above 59 indicates a tendency toward a morning type, and a score between 42 and 58 indicates an intermediate type. The overall reliability coefficient of the Chinese version of the MEQ exceeds 0.7 (36).

#### 2.3.5 The 17-item Hamilton Depression Rating Scale

The HAMD-17 is an observer-rated scale specifically designed to measure the intensity of depressive symptoms (37). This scale primarily employs a 5-point scale, with most items scoring from 0 to 4 points. However, some items use a 3-point scale, with a scoring range of 0 to 2 points. A HAMD-17 score between 7 and 17 indicates that the patient may exhibit symptoms of depression; a score between 17 and 24 clearly indicates the presence of depressive symptoms in the patient. The higher the score, the more severe the depressive symptoms and affective disorders are. The overall reliability coefficient of the Chinese version of HAMD-17 is 0.714 (38).

#### 2.3.6 The Hamilton Anxiety Rating Scale

The scale is designed to assess the level of anxiety symptoms in patients (39). It comprises a total of 14 questions, each rated on a 5-point scale ranging from 0 to 4 points. The overall score ranges from 0 to 54 points. A score of over 7 on the HAMA indicates the presence of anxiety symptoms; a score between 8 and 14 indicates that the patient may have mild anxiety symptoms; a score of 15 to 23 indicates the presence of moderate anxiety symptoms in the patient; and a score exceeding 24 points indicates severe anxiety symptoms. The total reliability coefficient of the Chinese version of HAMA ranges from 0.83 to 1.00 (40).

#### 2.3.7 The Montreal Cognitive Assessment

The primary purpose of this scale is to evaluate cognitive impairment, covering seven key cognitive domains: visual construction skills, executive function, memory, language, attention-concentration, calculation, abstract thinking, and orientation (41). The range of the overall score is set from 0 to 30 points, and if the MoCA score is below 26 points, it indicates the presence of cognitive impairment. The overall reliability coefficient of the Chinese version of MoCA is 0.857 (42).

### 2.4 Statistical analysis

All the statistical analyses were conducted using IBM SPSS Statistics Version 25.0 software and GraphPad Prism software. Initially, the study examined the differences between the two variable groups (the group with cognitive impairment and the group without cognitive impairment). The chi-square (χ2) test was used to compare categorical variables, which were expressed in terms of counts (percentages). The study also applied the Kolmogorov–Smirnov (K–S) test to preliminarily assess the normal distribution of continuous variables. For variables that followed a normal distribution, the mean ± standard deviation was utilized to describe them, and the independent sample t-test was employed to compare the differences between the groups. For variables with a non-normal distribution, the median (interquartile range) was used for description, and the Mann–Whitney U test was used to analyze inter-group differences. Secondly, the study further utilized Spearman correlation analysis to explore the correlation between the MoCA scores and scores from other related scales. In the binary logistic regression analysis, the presence or absence of cognitive impairment was used as the dependent variable, and statistically significant related features were selected as independent variables to construct a binary logistic regression model. To evaluate the effectiveness of each factor, the study also plotted receiver operating characteristic (ROC) curves and calculated the area under the curve (AUC) to determine its diagnosis ability.

## 3 Results

### 3.1 Demographic differences between MDD patients with or without cognitive impairment hospitalized during the acute phase

According to the inclusion and exclusion criteria, a total of 126 patients were included in the study. Of these, 80 (63.49%) were diagnosed with MDD and cognitive impairment. The participants were divided into two groups: the cognitive impairment group consisted of 80 patients, including 56 men and 24 women; the group without cognitive impairment consisted of 46 patients, with 26 men and 20 women. The mean age of the cognitive impairment group was 37.5 years (22, 53.75), while the mean age of the non-cognitive impairment group was 26.5 years (19, 37.75). The age difference between the two groups was statistically significant (*P* < 0.05). Additionally, there were significant differences between the two groups in terms of education level and place of residence (*P* < 0.05). However, no significant differences were observed between the two groups in terms of sex, marital status, BMI, comorbidities, or bed partner (*P* > 0.05) (**Table 1**).

**Table 1:** Demographics of MDD patients with or without cognitive impairment hospitalized during the acute phase.

Variable	With cognitive impairment (n=80)	Without cognitive impairment (n=46)	*t-value*	*P-value*
Age (years)	37.5 (22,53.75)	26.5 (19,37.75)	-3.193	<0.001
Sex				
Male, n (%)	56 (70)	26 ( 56.5)	2.335	0.127
Female, n (%)	24 (30)	20 (43.5)		
Education				
Middle school or less, n (%)	13 (16.3)	1 (2.2)	9.904	<0.05
Junior high school, n (%)	8 (10)	6 (13)		
High school, n (%)	30 (37.5)	12 (26.1)		
College or more, n (%)	29 (36.3)	27 (58.7)		
Marital status				
Unmarried, n (%)	26 (25.5)	27 (58.7)	5.429	0.066
Married, n (%)	30 (29.4)	18 (39.1)		
Divorced, n (%)	46 (45.1)	1 (2.2)		
BMI				
<18.5	12 (15)	8 (17.4)	1.095	0.798
18.5-24	43 (53.8)	27 (58.7)		
24-28	18 (22.5)	7 (15.2)		
>28	7 (8.8)	4 (8.7)		
Any other diseases				
No, n (%)	31 (38.8)	20 (56.5)	3.724	0.054
Yes, n (%)	49 (61.3)	26 (43.5)		
Rural/urban Residence				
Rural, n (%)	48 (60)	18 (39.1)	5.1	<0.05
Urban, n (%)	32 (40)	28 (60.9)		
Bed partner				
No, n (%)	21 (26.3)	24 (52.2)	8.549	0.057
Yes, n (%)	59 (73.8)	22 (47.8)		

BMI, body mass index.

### 3.2 Clinical correlation factor differences between MDD patients with or without cognitive impairment hospitalized during the acute phase

In terms of sleep quality and sleep cognition, no significant differences were observed between the two groups. The PSQI (*P* > 0.05), PSAS (*P* > 0.05), HAMA (*P* > 0.05), and DBAS (*P* > 0.05) scores were all lower than normal values. When comparing the circadian rhythm types of the two groups, the results showed that the intermediate type was the most common, followed by the morning and evening types, but there was no significant difference between the two groups (*P* > 0.05). However, there was a statistically significant difference in the HAMD-17 scores between the two groups, with the cognitive impairment group having significantly higher HAMD-17 scores than the non-cognitive impairment group (*P* < 0.05) (**Table 2**).

**Table 2:** Clinical correlation factors of MDD patients with or without cognitive impairment hospitalized during the acute phase.

Variable	With cognitive impairment (n=80)	Without cognitive impairment (n=46)	*t-value*	*P-value*
PSQI score	14 (11,17)	15 (11,17.25)	-0.419	0.675
Sleep quality	2 (2,3)	2 (1,3)	-0.108	0.914
Sleep latency	3 (2.25,3)	3 (2,3)	-0.348	0.728
Sleep time	2 (1,3)	2 (1,3)	-0.492	0.623
Sleep efficiency	3 (1,3)	3 (2,3)	-1.142	0.254
Sleep disturbances	1 (1,1)	1 (1,1)	-0.693	0.488
Sleep medications	2 (0,3)	3 (0,3)	1.103	0.270
Daytime dysfunction	3 (2,3)	3 (2,3)	0.811	0.417
PSAS score	41.36 ± 11.36	42.02 ± 12.05	-0.307	0.760
DBAS -16 score	37 (30.25,44.25)	36 (30.75,43.25)	-0.355	0.722
MEQ				
Morning type, n (%)	23 (28.7)	19 (41.3)	2.573	0.276
Intermediate type, n (%)	40 (50)	21 (45.7)		
Evening type, n (%)	17 (21.3)	6 (13)		
HAMD-17 score	22 (18,25.75)	19 (13,22.25)	-2.799	<0.01
HAMA score	20.16 ± 6.77	17.93 ± 7.25	1.733	0.086

PSQI, Pittsburgh Sleep Quality Index; PSAS, Pre-sleep Arousal Scale; DBAS-16, 16-item Dysfunctional Beliefs and Attitudes about Sleep Scale; MEQ, Morningness-Eveningness Questionnaire; HAMD-17, 17-item Hamilton Depression Rating Scale; HAMA, Hamilton Anxiety Rating Scale.

### 3.3 Correlation analysis of cognitive function and other parameters in MDD patients hospitalized during the acute phase

**Figure 1:**
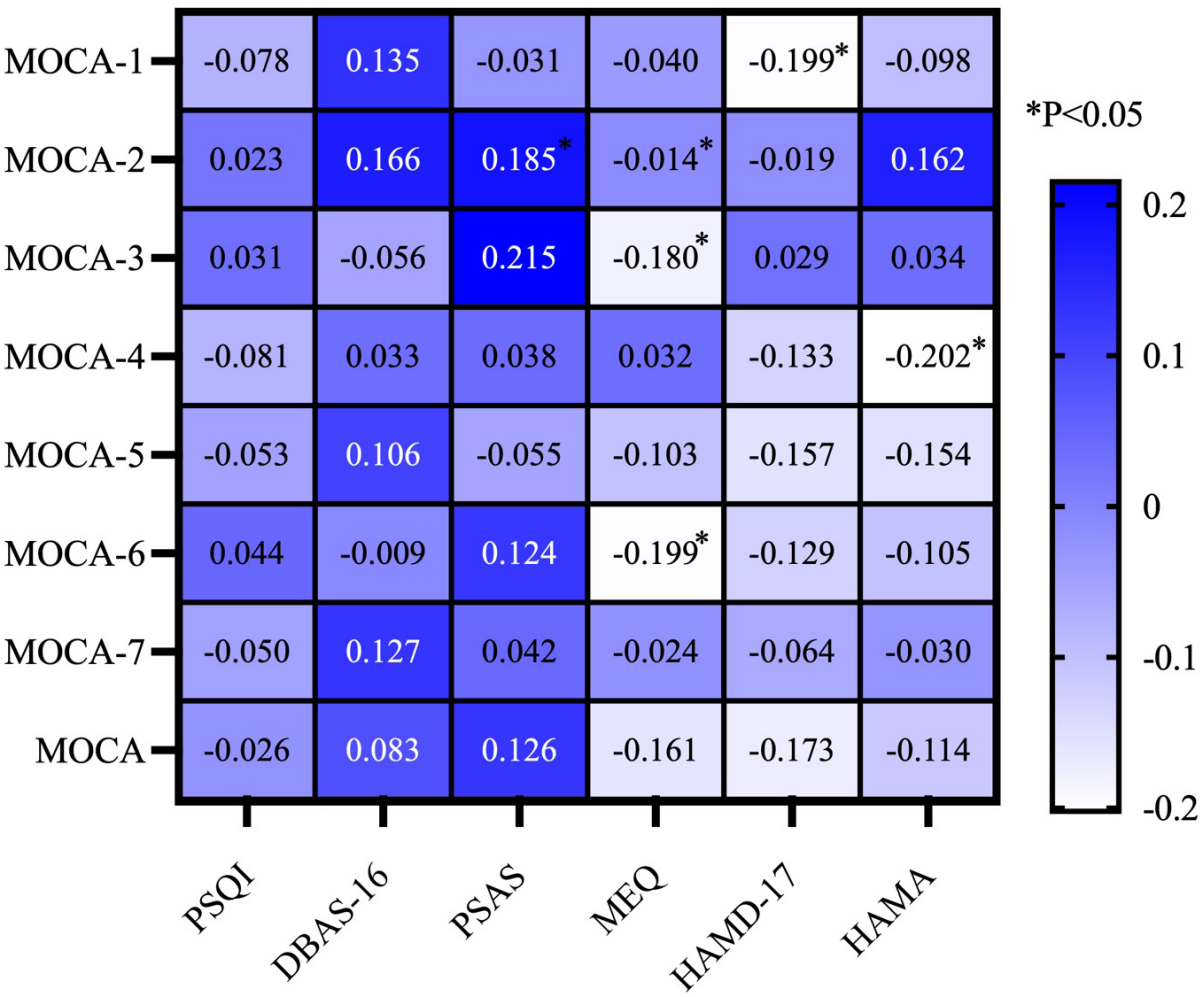
Correlation analysis of cognitive function and other parameters in MDD patients hospitalized during the acute phase. MOCA-1, Visuospatial and Executive; MOCA-2, Naming; MOCA-3, Memory; MOCA-4, Attention; MOCA-5, Language; MOCA-6, Abstraction; MOCA-7, Orientation; MoCA, Montreal Cognitive Assessment; PSQI, Pittsburgh Sleep Quality Index; HAMD-17, 17-item Hamilton Depression Rating Scale; HAMA, Hamilton Anxiety Rating Scale; PSAS, Pre-Sleep Arousal Scale; DBAS-16, 16-item Dysfunctional Beliefs and Attitudes about Sleep Scale; MEQ, Morningness-Eveningness Questionnaire.

To investigate the relationship between cognitive function and other variables, we conducted a correlation study. In this study, we employed Spearman correlation analysis on continuous numerical data. The Spearman correlation analysis revealed a significant positive correlation between the PSAS score and naming ability (*P* < 0.05). Concurrently, there was a significant negative correlation between the MEQ score and abilities in naming, memory, and abstraction (*P* < 0.05). The HAMD score showed a negative correlation with visuospatial and executive ability (*P* < 0.05). Additionally, the HAMA score was positively correlated with attention (*P* < 0.05) (**Figure 1**).

### 3.4 Correlation factors of cognitive impairment based on a binary logistic model in MDD patients hospitalized during the acute phase

After completing the correlation analysis, we further utilized a binary logistic regression model (backwards: Wald) with cognitive impairment as the outcome variable to identify different independent variables and their associations with the univariate analysis. The research results indicated that in the binary logistic regression model, a high HAMD-17 score (OR = 1.102, 95% CI = 1.03-11.79, *P* < 0.01), older age (OR = 1.049, 95% CI = 1.013-1.087, *P* < 0.01), and living in rural areas (OR = 2.7, 95% CI = 1.083-6.731, *P* < 0.05) were significantly associated with cognitive impairment in MDD patients (**Table 3**).

**Table 3:** Correlation factors of cognitive impairment based on a binary logistic model in MDD patients hospitalized during the acute phase.

Variable	B	SE	Wald X^2^	*P*	OR (95%CI)
HAMD-17 score	-0.98	0.034	8.074	<0.01	1.102 (1.031-1.179)
Education					
Middle school or less	1.234	1.187	1.080	0.299	3.434 (0.335-35.166)
Junior high school	-0.905	0.75	1.455	0.228	0.405 (0.093-1.760)
High school	0.515	0.482	1.142	0.285	0.726 (0.651-4.306)
College or more	-	-	-	-	-
Age (years)	0.048	0.018	7.140	<0.01	1.049 (1.013-1.087)
Rural/Urban Residence					
No	0.993	0.466	4.541	<0.05	2.7 (1.083-6.731)
Yes	-	-	-	-	-

HAMD-17, 17-item Hamilton Depression Rating Scale.

### 3.5 ROC analysis of the factors that influence cognitive impairment in MDD patients hospitalized during the acute phase

**Figure 2:**
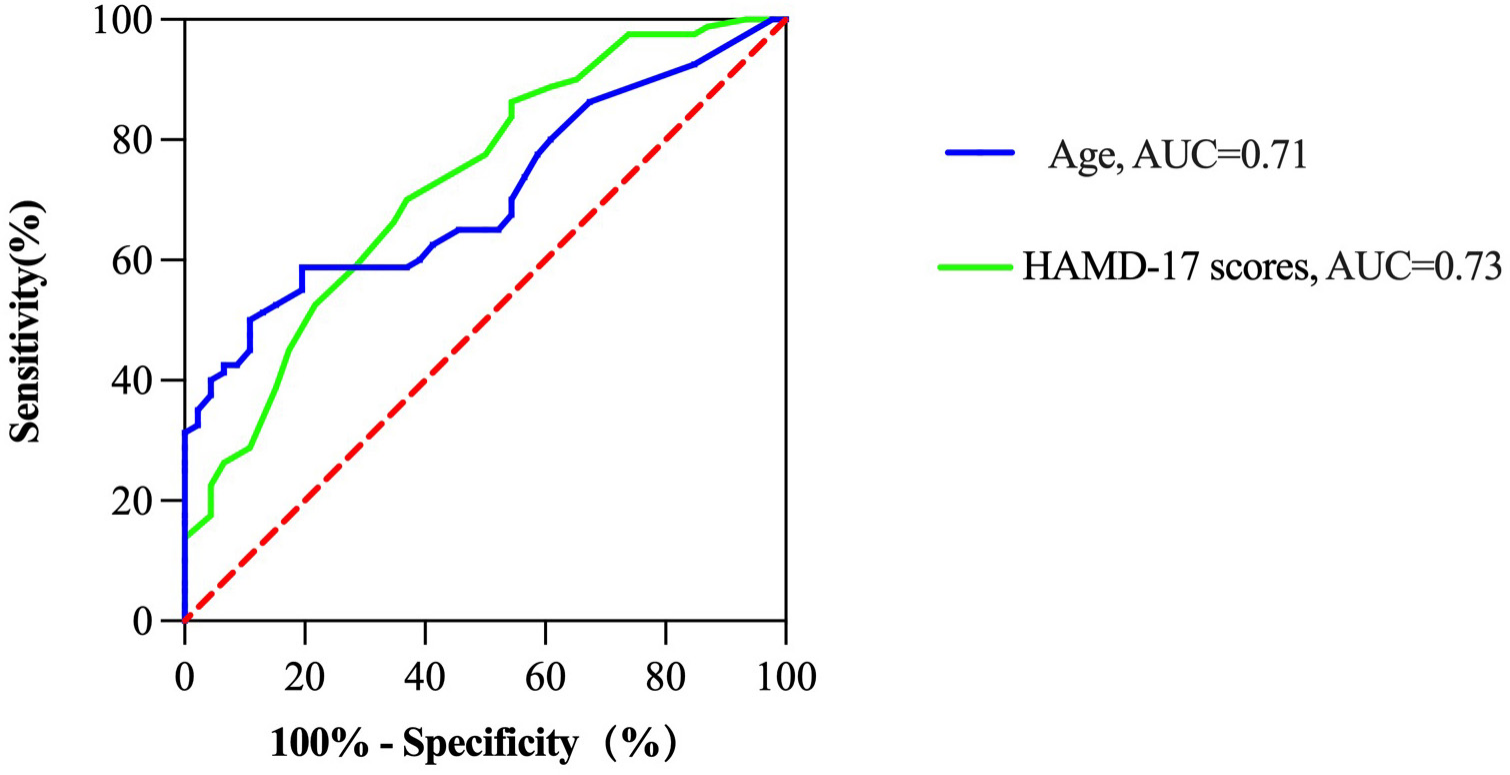
ROC analysis of the factors that influence cognitive impairment in MDD patients hospitalized during the acute phase.

We conducted ROC analysis to evaluate the factors identified in the binary logistic regression analysis that were significantly associated with cognitive impairment in MDD patients (**Table 4**). The results of the ROC curve analysis revealed that age and the HAMD-17 score were significantly associated with cognitive impairment in MDD patients (*P* < 0.001). Specifically, the ROC analysis results for age revealed an AUC of 0.71, a cut-off value of 39.5, a sensitivity of 59%, and a specificity of 80%. For the HAMD-17 score, the ROC analysis showed an AUC of 0.73, a cut-off value of 19.5, a sensitivity of 70%, and a specificity of 63% (**Figure 2**).

**Table 4:** ROC analysis of the factors that influence cognitive impairment in MDD patients hospitalized during the acute phase.

	AUC	Cut-off	Youden index	Sensitivity	Specificity
HAMD-17 score	0.73	19.5	0.33	0.7	0.63
Age (Years)	0.71	33.5	0.392	0.59	0.80

HAMD-17, 17-item Hamilton Depression Rating Scale; AUC, Area Under the Curve

## 4 Discussion

The aim of this study was to investigate the prevalence and clinical relevance of cognitive impairment in MDD patients hospitalized during the acute phase. The main findings of the study are as follows: (i) The prevalence of cognitive impairment among MDD patients hospitalized during the acute phase was 63.49%. (ii) Among MDD patients hospitalized during the acute phase, there were significant differences in age, education level, place of residence, and clinical symptoms (HAMD-17 score) between patients with cognitive impairment and those without cognitive impairment. (iii) The study also found that visuospatial and executive abilities were negatively correlated with the HAMD-17 score; naming ability was positively correlated with pre-sleep arousal level; memory was negatively correlated with the MEQ score; attention was negatively correlated with the HAMA score; and abstraction was negatively correlated with the MEQ score. (iv) Further analysis showed that the HAMD-17 score, age, and place of residence were important clinically relevant factors for cognitive impairment in MDD patients hospitalized during the acute phase. (v) By plotting the ROC curve, it was found that the AUC of the HAMD-17 score was approximately 73%, while the AUC of age was approximately 71%. This study explored for the first time the correlation between sleep characteristics and cognitive function in MDD patients hospitalized during the acute phase and provided a detailed analysis of factors such as the degree of wakefulness before bedtime, sleep cognition, and circadian rhythm types. In addition, we conducted in-depth research on the accuracy of the HAMD-17 score and age in identifying cognitive impairment in MDD patients hospitalized during the acute phase.

First, we reported that the prevalence of cognitive impairment among MDD patients hospitalized during the acute phase was 63.49%. Compared to a cross-sectional study of the elderly population in China that found a prevalence of depression among the elderly to be 15.9%, with 36.4% of MDD patients suffering from mild cognitive impairment (MCI), our study identified a lower prevalence of cognitive impairment at 63.49% (43). However, most patients in our study had a history of antidepressant use and hospitalization, and the definition of MDD was stricter, which may have excluded more patients from the study. Another 3-year prospective study revealed that during the acute phase of MDD, the prevalence of cognitive impairment, energy deficiency, and sleep problems ranged from 85% to 94% (27). Although there are differences in the above research results, they all indicate that patients with depression have a higher incidence of cognitive impairment, consistent with the results of our study. The discrepancies in results may stem from the heterogeneity of the related research, which could be influenced by demographic characteristics, age, and socio-cultural features. Nevertheless, these studies largely support our findings, indicating a relatively high incidence of cognitive impairment in MDD patients hospitalized during the acute phase.

Second, this study revealed significant disparities in clinical characteristics (such as age, education level, and place of residence) between MDD patients with or without cognitive impairment who were hospitalized during the acute phase. Existing research suggests that MDD patients hospitalized for cognitive impairment during the acute phase are typically older and have lower levels of education compared to those without cognitive impairment. In addition, the majority of MDD patients with cognitive impairment who were hospitalized during the acute phase reside in rural areas. Previous studies have emphasized that age remains the most critical factor contributing to cognitive decline, while insufficient early education may increase the risk of cognitive deterioration in patients in their later years (44). A study employed magnetic resonance imaging technology to investigate variations in brain structure and cognitive function in different age groups, finding that both cross-sectional and longitudinal studies observed a significant non-linear 1% annual decrease in cerebral cortex thickness before the age of 14 and after the age of 60 (45). This trend was particularly evident in the frontal and parietal cortical regions, responsible for executive function and attention (46). However, current research exploring the influence of education level on cognitive impairment in MDD patients is limited. A study on the memory and processing speed of initial and untreated depression patients showed that education level had a more significant confounding effect compared to the severity of depression (47). Moreover, a large-scale cross-sectional study of the Chinese population revealed that the cognitive function of the elderly residing in eastern China or urban areas seemed to be better than that of the elderly living in central and western China or rural areas. This finding suggests that residential areas may also have subtle effects on cognitive function (48).

Third, the evaluation results of multiple clinically relevant scales (such as PSQI, PSAS, DBAS-16, MEQ, HAMD-17, and HAMA) indicate that among hospitalized MDD patients in the acute phase, those with cognitive impairment exhibited higher scores on the PSQI, PSAS, DBAS-16, HAMD-17, and HAMA compared to those without cognitive impairment. Additionally, according to the MEQ results, individuals with cognitive impairment had a higher proportion of morning chronotypes, while among MDD patients with a late chronotype, the proportion with cognitive impairment was lower than that of those without cognitive impairment. We investigated the correlation between various sleep characteristics and cognitive impairment in MDD patients hospitalized during the acute phase. Using MoCA scoring, we observed significant but relatively weak correlations between certain cognitive functions and the PSAS, MEQ, HAMD-17, and HAMA scores. However, no significant correlation was observed between other scales, or the correlation was too weak to have practical significance. Previous studies have shown that sleep quality is a risk factor for depression and has an independent correlation (49). Factors such as high alertness before falling asleep, incorrect sleep cognition, and disrupted circadian rhythms can exacerbate the decline in sleep quality. For instance, a clinical study revealed that incorrect sleep cognitive concepts were more likely to lead to sleep disorders in patients with mental disorders. In this study, the authors proposed that addressing these incorrect sleep concepts was key to improving sleep problems (6). Another review showed that after adjusting for covariates such as demographics, there was still a significant correlation between sleep quality and cognitive impairment in MDD patients, but no such correlation was found in healthy participants (50). These research results show that a long-term decline in sleep quality may lead to cognitive impairment. Although this trend was not significant in our study, if it persists, it may impact patients’ treatment plans and management.

Fourth, in this study, we reported three important related factors: HAMD score, age, and education level. The results indicated that all three factors might have an impact on cognitive function during acute hospitalization. This discovery was consistent with previous research findings. From a psychopathological perspective, in patients with MDD, the gray matter volume of the left amygdala, bilateral anterior cingulate cortex (PreC), and posterior cingulate cortex (PCC) increased, while the gray matter volume of the right lower parietal lobe decreased. The changes in these brain regions were closely related to cognitive control, maintenance of episodic memory, and attention, and the integration and conflict resolution of perceptual information (51). In addition, current research has not only focused on changes in a single brain region but also delved into the activation patterns of neural circuits between different regions of the brain. According to functional magnetic resonance imaging (fMRI) studies, the functional connectivity (FC) patterns between brain regions in patients with depression might have been related to cognitive function. For instance, the study by Zhang et al. found that compared to healthy individuals, patients with depression exhibited higher FC between the right dorsolateral prefrontal cortex (DLPFC) and regions such as the left inferior temporal gyrus, left cingulate cortex, and right inferior frontal gyrus (52). The frontal cortex plays a crucial role in the brain, involving language, cognition, executive function, and emotional regulation (53). Experts believe that the enhanced FC between the thalamus and DLPFC might be a cause of cognitive decline, as the thalamus plays a crucial role in cognitive processes (54). Additionally, another study found a positive correlation between the HAMD score and the resting state functional connectivity (rsFC) of the right DLPFC (55). This finding suggests a certain correlation between the severity of depression and the strength of functional connections between these critical brain regions.

Finally, the ROC curve was plotted. The AUC for age and the HAMD-17 score were approximately 71% and 73%, respectively. These results indicate that age and the severity of depressive symptoms had certain diagnostic value in evaluating whether MDD patients hospitalized during the acute phase exhibited cognitive impairment.

Our research had several limitations. First, as a cross-sectional study, we were unable to establish a causal relationship between cognitive impairment and other factors among hospitalized MDD patients during the acute phase. Second, since our sample was confined to Chinese individuals, the applicability of our findings to populations in other countries may be limited due to sociodemographic traits. Third, our sample predominantly consisted of hospitalized patients who were typically in the acute phase of the disease, implying that our findings may not be generalizable to MDD patients during the stable phase. Additionally, the PSQI score relies on a patient’s subjective experiences, potentially introducing individual cognitive biases. Moreover, the research period extending over a month might result in recall bias. Consequently, it is essential to conduct in-depth cohort studies in the future to further validate our findings.

In summary, this study indicates that the prevalence of cognitive impairment is higher among MDD patients hospitalized during the acute phase. Factors such as a higher HAMD-17 score, older age, and lower education levels are associated with cognitive impairment in these patients. Additionally, the HAMD-17 score and age can also predict the severity of cognitive impairment. These findings suggest that swiftly improving the clinical condition of high-risk populations may help reduce their risk of cognitive impairment. Therefore, clinicians should conduct detailed evaluations based on the individual circumstances of each patient and closely monitor the cognitive function of those with depression. Given the mutual effect between depressive symptoms and cognitive function, the timely assessment of the cognitive status of patients with depression is crucial for early behavioral intervention during the acute phase.

## Data Availability

The original contributions presented in the study are included in the article. Further inquiries can be directed to the corresponding author.
